# Stratified Evaluation of GPT’s Question Answering in Surgery Reveals Artificial Intelligence (AI) Knowledge Gaps

**DOI:** 10.7759/cureus.48788

**Published:** 2023-11-14

**Authors:** Rebecca Murphy Lonergan, Jake Curry, Kallpana Dhas, Benno I Simmons

**Affiliations:** 1 Department of Medical Education, Chelsea and Westminster Hospital NHS Foundation Trust, London, GBR; 2 Centre for Ecology and Conservation, University of Exeter, Penryn, GBR

**Keywords:** artificial intelligence in medicine, artificial intelligence in surgery, surgery, large language models, chatgpt

## Abstract

Large language models (LLMs) have broad potential applications in medicine, such as aiding with education, providing reassurance to patients, and supporting clinical decision-making. However, there is a notable gap in understanding their applicability and performance in the surgical domain and how their performance varies across specialties. This paper aims to evaluate the performance of LLMs in answering surgical questions relevant to clinical practice and to assess how this performance varies across different surgical specialties.

We used the MedMCQA dataset, a large-scale multi-choice question-answer (MCQA) dataset consisting of clinical questions across all areas of medicine. We extracted the relevant 23,035 surgical questions and submitted them to the popular LLMs Generative Pre-trained Transformers (GPT)-3.5 and GPT-4 (OpenAI OpCo, LLC, San Francisco, CA). Generative Pre-trained Transformer is a large language model that can generate human-like text by predicting subsequent words in a sentence based on the context of the words that come before it. It is pre-trained on a diverse range of texts and can perform a variety of tasks, such as answering questions, without needing task-specific training. The question-answering accuracy of GPT was calculated and compared between the two models and across surgical specialties. Both GPT-3.5 and GPT-4 achieved accuracies of 53.3% and 64.4%, respectively, on surgical questions, showing a statistically significant difference in performance. When compared to their performance on the full MedMCQA dataset, the two models performed differently: GPT-4 performed worse on surgical questions than on the dataset as a whole, while GPT-3.5 showed the opposite pattern. Significant variations in accuracy were also observed across different surgical specialties, with strong performances in anatomy, vascular, and paediatric surgery and worse performances in orthopaedics, ENT, and neurosurgery.

Large language models exhibit promising capabilities in addressing surgical questions, although the variability in their performance between specialties cannot be ignored. The lower performance of the latest GPT-4 model on surgical questions relative to questions across all medicine highlights the need for targeted improvements and continuous updates to ensure relevance and accuracy in surgical applications. Further research and continuous monitoring of LLM performance in surgical domains are crucial to fully harnessing their potential and mitigating the risks of misinformation.

## Introduction

Large language models (LLMs) are artificial intelligence (AI) models designed for language processing tasks requiring the analysis of text-based data [1]. The utility of LLMs lies in their capacity to process and analyse vast quantities of data and generate coherent and contextually relevant outputs. As such, they promise to revolutionise how humans access knowledge and answer questions. Due to their diverse capabilities and applications, recent attention has focused on the development and training of LLMs, capable of performing a diverse array of tasks, over contextualised language models (CLMs) tailored to specific topics or tasks [2]. The general public is also increasingly interested in utilising LLMs, like Chat Generative Pre-trained Transformer (ChatGPT), which have made them part of the zeitgeist.

Large language models have broad potential medical and surgical applications for doctors, patients, and students [3]. These include an academic resource or study tool for triaging presentations, supporting clinical decision-making, and addressing patient concerns [1]. Moreover, as LLMs continue to improve their chain-of-thought reasoning skills, their capabilities and potential roles will continue to increase [4]. There is intense research interest in assessing and validating LLMs for use in medicine. For example, GPT has succeeded in tasks requiring broad knowledge, such as passing the United States Medical Licensing Examination (USMLE) [5], as well as aiding in specific tasks, such as specialised multi-disciplinary team decisions [6]. However, despite this recent interest, we identify two gaps in the medical LLM literature.

First, the use of LLMs in surgery is less understood [3]. However, LLMs are a good fit for the surgical specialty [3]. Surgery, compared to other medical fields, celebrates decisive, binary decision-making (typically whether or not to operate), which aligns well with the capabilities of artificial intelligence. Surgeons also work within a narrower field than medics, rendering their clinical work more predictable, both in its range of conditions and its management strategies, which are more formulaic [7]. The surgical profession has a great deal to gain from AI assistance, which could support surgeons’ clinical practice, allowing them to prioritise theatre time [8,9].

The second gap is that existing work evaluating LLM performance on medical question-and-answer datasets tends to assess accuracy across the dataset as a whole, masking any variation present in relevant subgroups of the data, such as medical specialty [4,10]. This is despite evidence from one of the few studies to examine subgroup performance that model performance differs across subject areas [11,12]. The lack of consideration of subgroup-level accuracies hinders efforts towards improving LLMs through targeted enhancements. Calculating accuracies for different subgroups would allow the identification of specific areas where misinformation is more common, offering a more nuanced understanding of a model’s strengths and weaknesses. In turn, this would allow the evaluation of risk and help guide models’ future development.

Presently, there is limited published research on AI’s ability to answer surgical questions and no stratified evaluations of LLMs on surgical subspecialties. Here, we explore the performance of the LLM GPT in answering surgical questions. GPT is one of the most well-known and easily accessible LLMs to medical professionals and the general public due to its conversational interface, ChatGPT [13]. To maximise real-world applications and relevance to clinical practice, we assessed the performance of GPT on data derived from legitimate medical examinations undertaken by clinicians. We find that GPT performs less well on surgical questions than medical questions and find variations in question-answering accuracy between surgical subspecialties. We discuss the implications of these findings for LLMs’ role in surgical practice today and in the future.

## Materials and methods

**Figure 1:**
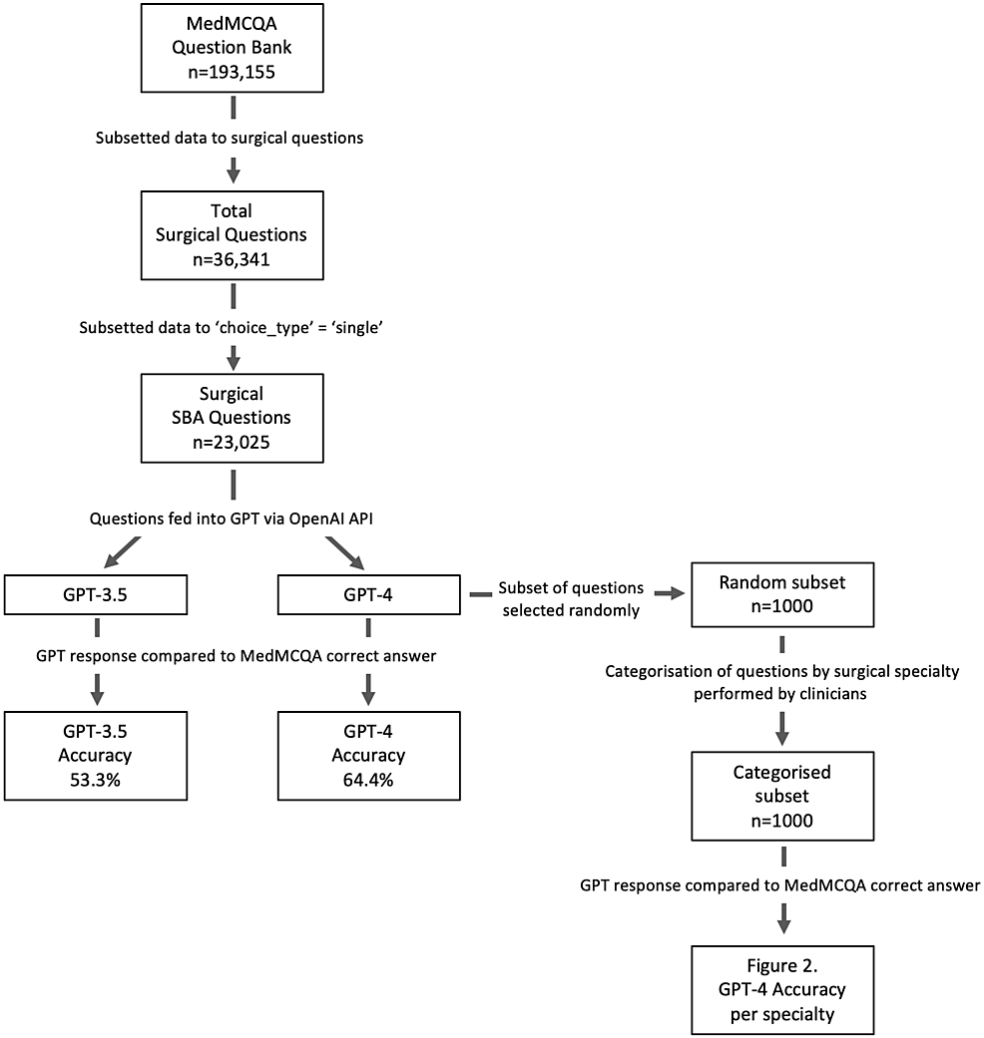
Flow diagram of the study methodology Flow diagram showing the study methodology from data subsetting to analysis. GPT: Generative Pre-trained Transformer; SBA: single best answer

The study was conducted at Chelsea and Westminster Hospital in London, UK. An overview of our methodology is summarised in Figure 1.

We selected MedMCQA as our question bank, a publicly available large-scale multi-choice question answering (MCQA) dataset for AI testing consisting of clinically-based questions covering 21 diverse subject areas [11]. Questions are taken from the All India Institute of Medical Sciences (AIIMS) and National Eligibility cum Entrance Test (NEET) postgraduate entrance exams, which test the professional knowledge of graduate medical students holding a medical degree for admission to postgraduate medical training in India. The dataset consists of 193,155 multiple-choice questions, collated and validated by a range of expert contributors. A detailed explanation of the correct answer is provided by a clinician, distinguishing MedMCQA from other leading datasets. MedMCQA provides a reliable benchmark for the assessment of LLM performance and has been successfully used to compare the performances of multiple LLMs and human candidates [4,14].

Of the 21 categories of questions in MedMCQA, four relate to the clinical practice of surgery and correlate with the knowledge of surgeons: surgery (general), ear, nose, and throat (ENT), orthopaedics, and anatomy. We therefore filtered MedMCQA into these four question categories. This reduced the size of the dataset to 36,341 questions. We then subsetted the data to questions where the ‘choice_type’ was ‘single’. There is only one other ‘choice_type’ in the dataset, which is ‘multi’. ‘Single’ refers to questions where there is a single best answer; ‘multi’ refers to questions where there are multiple correct answers. We chose to focus on 'single', as only a single correct answer is provided by the MedMCQA question bank for each question. This reduced the dataset to 23,035 questions.

Questions were fed to GPT via OpenAI’s API (Application Programming Interface) OpenAI, LLC, San Francisco, CA) using the OpenAI Python package. Note that this is a zero-shot method; the respective LLMs have no prior training or fine-tuning for the task at hand. To prevent possible data leakage, only the question, possible answers, and question ID were submitted to the OpenAI API. We submitted questions to two LLMs: GPT-3.5 and GPT-4. We asked each model for a letter-only response to questions (i.e., for the LLM to provide the correct answer only).

A random set of 1,000 MedMCQA questions and answers generated by GPT-4 were extracted from the dataset. Of this subset, question content was manually categorised into surgical specialties by two independent clinician reviewers. Surgical specialty categorisations were based on the UK Surgical Royal Colleges’ surgical specialties. The two reviewers are practicing doctors in the UK, with clinical experience in medicine and surgery and specialist knowledge in medical education and assessment. Following review by both clinicians, categorisation was compared, and, where there was discordance, a collective agreement was reached to give a standardised outcome. While some questions within the MedMCQA database had been assigned a subtopic, this field was sporadically completed and was non-standardised; therefore, the existing subtopic field on MedMCQA was disregarded.

Overall accuracy measures for GPT-3.5 and GPT-4 were calculated as percentage accuracy (total number of correctly answered questions divided by the total number of questions) and compared to the recently published zero-shot accuracy of GPT-3.5 and GPT-4 on the MedMCQA dataset as a whole, which is 50.1% and 69.5%, respectively [2]. To assess whether there were statistically significant differences in accuracy between GPT-3.5 and GPT-4, we used a two-proportion z-test. To assess whether there were statistically significant differences in accuracy between our results using GPT to answer surgical questions and published results of the accuracy of GPT on the MedMCQA dataset as a whole, we again used a two-proportions z-test. To assess whether there were statistically significant differences in accuracies between surgical specialties on the 1000-question subset, we used a chi-square goodness of fit test to test the assumption that the distribution of accuracy values is not equal between specialties. Statistical tests were carried out using the 'stats' package built into R version 4.3.0 (The R Core Team, R Foundation for Statistical Computing, Vienna, Austria) [15].

## Results

Of 23,025 surgical multiple-choice questions (MCQs), the zero-shot results for GPT-3.5 and GPT-4 were 53.3% and 64.4%, respectively. These accuracies were significantly different (X^2 ^= 585.53, df = 1, p-value < 0.01).

We found statistically significant differences in accuracy between our results using GPT-3.5 to answer surgical questions (53.3% accurate) and published results of the accuracy of GPT-3.5 on the MedMCQA dataset as a whole (50.1% accurate) (X2 = 84.174, df = 1, p-value < 0.01). We also found statistically significant differences in the accuracy between our results using GPT-4 to answer surgical questions (64.4% accurate) and published results of the accuracy of GPT-4 on the MedMCQA dataset as a whole (69.5% accurate) (X^2^ = 249.82, df = 1, p-value < 0.01).

**Figure 2:**
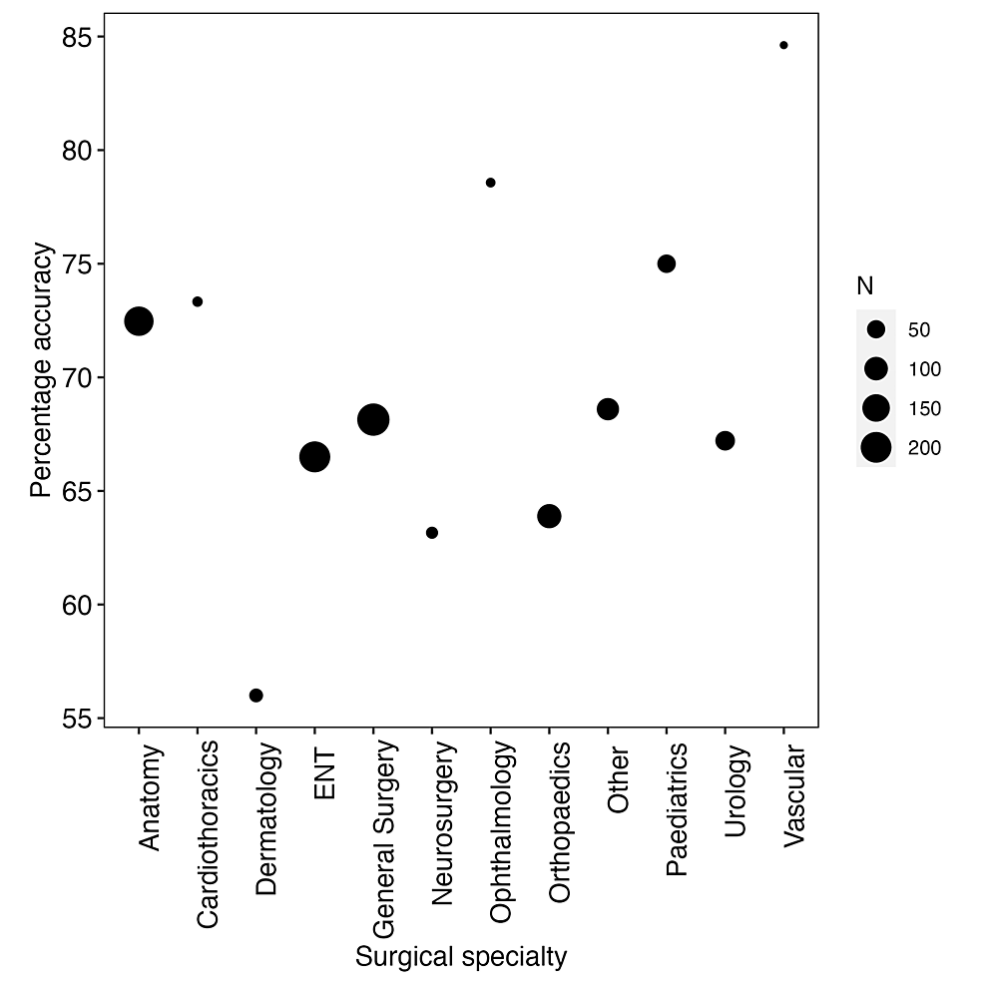
The variations in GPT-4's question-answering accuracy across surgical specialties Accuracy for each surgical specialty on the 1000-question GPT-4 subset; points are drawn proportional to N, where N is the number of questions for a given subspecialty. GPT: Generative Pre-trained Transformers

In the 1,000-question GPT-4 subset, the accuracy rates of GPT-4 between specialties are shown in Figure 2.

We found there was a significant difference in accuracy between specialties (X^2^ = 554.05, df = 11, p-value < 0.01). We found strong performances in anatomy, vascular, and paediatric surgery, but worse performances in orthopaedics, ENT, dermatology, and neurosurgery.

## Discussion

Here, we evaluated the ability of large language models to accurately answer clinically relevant questions about surgery and assessed how model performance varies between surgical specialties. We found that, overall, 54.3% of surgical questions in the MedMCQA dataset were answered correctly using GPT-3.5, and 64.4% were answered correctly using GPT-4. These results were significantly different from those found when GPT was applied to the MedMCQA dataset as a whole, which encompasses all of medicine, not just surgery (GPT-3.5: 53.3% surgical vs. 50.1% whole dataset; GPT-4: 64.4% surgical vs. 69.5% whole dataset) [2]. We also found that question-answering accuracy varied significantly by surgical specialty, with higher accuracies for specialties like anatomy, vascular, and paediatric surgery and lower accuracies for specialties like ENT, neurosurgery, and orthopaedics.

These results suggest that LLMs, like GPT-4, can perform well in specialised fields, such as surgery, with a zero-shot approach that is without the need for domain-specific or task-specific training. The model's capacity to handle a wide range of queries and prompts demonstrates how rapidly LLMs are closing the gap with contextualised language models [16, 17]. Moreover, the strength of ChatGPT, in particular, is its user-friendly and accessible web interface, which makes it easy to integrate into medical practice and to be adopted by the public [13]. These factors make a compelling argument for developers to direct resources increasingly in favour of LLMs rather than domain-specific contextualised language models [10]. However, it is important to note that the performance of LLMs like GPT can be refined further with prompt tuning strategies (without the need for dataset-specific fine tuning) [10]. This was recently shown with the Med-PaLM LLM, which performed well on a variety of multiple-choice question datasets relative to human evaluation [10].

The significant improvement in performance between GPT-3.5 and GPT-4 could be credited to more extensive training data and enhanced training methodologies used to develop GPT-4, which contribute to the model’s improved language comprehension and contextualisation. Much like how, in the medical field, guidelines are constantly reviewed and updated, GPT’s knowledge base must consistently be refreshed to utilise emerging information. The need to supply sequential models with larger and more far-reaching datasets must be critically considered when referring to GPT’s medical and surgical knowledge; up-to-date knowledge is essential for informed surgical decision-making and practice, and delays between accepted changes in practice and updates to GPT training data could potentially be disastrous. Recent developments to provide ChatGPT with internet access through Microsoft’s Bing search engine could help resolve this by ensuring ChatGPT has access to current knowledge [18]. Equally, LLM training data, as is common across datasets in general, undoubtedly contain hidden biases [19, 20]. In medicine, this is of particular importance because bias in translational research affects health outcomes [21, 22]. We must therefore take responsibility for how biases within LLM training data will be reflected in the model’s output.

The latest model, GPT-4, had significantly lower performance on surgical questions compared to the MedMCQA dataset as a whole [2]. Restricted as GPT is by its training data, this result suggests there may be a scarcity of surgically themed training data compared to non-surgical specialties. Conversely, however, we found that the older model, GPT-3.5, performed better on surgical questions than the whole MedMCQA dataset. The reasons for this surprising relationship are hard to discern as GPT is not an open-source model and the specific changes in training data and model structure between GPT-3.5 and GPT-4 are not public. However, this could imply that, in GPT-4’s expanded training dataset, the proportion of medical data that are not related to surgery was substantially greater than that which is surgical, thereby decreasing the relative proportion of surgical data over non-surgical data. As we have only been able to compare changes between two versions of GPT (the only two versions OpenAI made public at the time of analysis), we cannot draw conclusions about whether this decrease in surgical performance following GPT development is a concerning trend. As newer versions of GPT are released, future research could repeat our analyses to help answer this question.

We found significant variability in model accuracy between surgical specialties, suggesting that some areas might be covered more than others in GPT’s training data. It is possible that this variance relates to how specialised a given field is and thus how much information is readily available; this could explain, for example, why GPT performs better on questions about general surgery than neurosurgery [23]. However, we note that variation in sample size between subgroups means that accuracy values should be interpreted with caution for specialties with smaller sample sizes (Figure 2). One interesting result is that of anatomy. Given that anatomy is a ubiquitous, relatively unspecialised field, it is likely to have greater representation in GPT's training data. Additionally, it is one of the largest sample sizes in the MedMCQA dataset. As such, it might be expected that GPT would perform well on anatomy questions. One explanation for the lower accuracy of anatomy is that, because of its relatively unspecialised status, there could be a larger amount of inaccurate data relating to anatomy in GPT’s training data. In contrast, training data related to more niche specialties may be more likely to have a higher proportion of professionally written materials. Future work could look into how LLMs with more restricted training sets would compare to our results.

It is important to note the limitations of our study. First, if there is a relationship between the question ‘choice_type’ and specialty, focusing on ‘single’ type questions could introduce a bias. While this is theoretically possible, we do not expect such a relationship a priori.

Second, for some specialties, we have a small sample size. Given the significant time cost of manually categorising questions, a random sample of 1,000 questions was the best approach for the analysis of surgical specialties. A uniform distribution of questions across specialties would ensure more certainty around accuracy scores. However, achieving this would require manually categorising many thousands of questions to ensure a sufficient number of ‘rare’ specialty questions were encountered. For example, if a rare specialty made up 1% of the questions, approximately 10,000 questions would have to be categorised in order to obtain 100 questions for just that specialty. Given that five specialties make up less than 5% of the data, this was infeasible for this study.

Third, the MedMCQA dataset contains questions targeted at recent graduates. However, some surgical specialties are more specialised than others; for example, a recent medical graduate would be expected to know more about general surgery than neurosurgery. This means that the questions in the dataset might require more advanced decision-making for general surgery than neurosurgery. Thoroughly testing more specialised areas, such as neurosurgery, may therefore require focused datasets designed specifically to test advanced knowledge in these more niche fields. However, we note that studies assessing LLM accuracy using the MedMCQA dataset are still relevant for testing LLMs' ability to reproduce the knowledge expected of more junior doctors.

Fourth, OpenAI recently released a new version of GPT (November 6, 2023), after our analysis was completed. This new version (GPT-4 Turbo) is a mid-generation update to GPT-4 rather than a major new release (i.e., it is not GPT-5), and so results are likely to not be substantially different from those presented here. However, it is possible that results may differ somewhat using this new version. We therefore encourage future research to benchmark GPT-4 Turbo to quantify its performance.

Fifth, LLMs are stochastic, and thus it is possible for GPT to respond differently to questions if asked on multiple occasions. In this study, we asked GPT each question once, as is common in other papers assessing LLM performance [10]. Future research gauging the variability in LLM responses to multiple instances of the same question is a potentially fruitful area.

Sixth, the comparison between our results and the published results in Toma et al.'s study [2] should be interpreted with two caveats. First, we cannot be sure of the exact sub-version of GPT used by the authors, other than the major release milestones of GPT-3.5 and GPT-4. OpenAI iterates on their models regularly, and thus it is possible that our questions were analysed with a slightly different version of GPT-3.5 or GPT-4 than those used by Toma et al. [2]. Second, the filtering of questions between ours and the comparison paper may be different. For example, the comparison paper may include questions with multiple best answers, which we exclude. If this is the case, it is unknown how much this would affect the comparability of our results, as it depends on whether there is a systematic difference in GPT’s ability to answer questions with single versus multiple best answers. Future research could help answer this question by comparing LLM performance on ‘single’ and ‘multi’ questions in the MedMCQA dataset.

## Conclusions

The value of LLMs in the time- and resource-strained setting of surgery lies in their ability to complete a myriad of tasks almost instantaneously, thus relieving pressure on the surgeon and enabling them to prioritise their craft specialty. Here we show the capabilities and limitations of GPT-3.5 and GPT-4 in addressing surgical questions. While both models showcased a good ability to interpret and apply surgical knowledge without specialised training, the performance varied significantly between different fields of surgery. This uneven proficiency underscores the need for more extensive and balanced training datasets to ensure consistency across surgical domains.

There are legitimate concerns regarding the premature use of LLMs in the patient-safety-oriented field of healthcare. While we endorse their ongoing development, we caution that LLMs must be truly ready for use in surgery, confirmed through accurate evaluation. We therefore advocate for robust validation through stratified evaluation, enabling targeted enhancements.
